# Immune Escape in Renal Cell Carcinoma: Latest Research and Treatment Strategies

**DOI:** 10.3390/ijms27073064

**Published:** 2026-03-27

**Authors:** Kaixiang Huang, Ke Chen

**Affiliations:** Department of Urology, Tongji Hospital, Tongji Medical College, Huazhong University of Science and Technology, Wuhan 430100, China

**Keywords:** renal cancer, immune escape, MHC-1, PD-L1, VHL

## Abstract

Renal cancer is a common malignant tumor in the urinary system. Current research has found that immune escape in kidney cancer can prevent immune system attacks through specific mechanisms, making it difficult for the immune system to effectively kill cancer cells, and promote the progression and metastasis of kidney cancer. Therefore, despite the continuous updating of immunotherapy methods for kidney cancer, the high recurrence rate, high drug resistance, and severe side effects of kidney cancer patients are still difficult to solve. This review systematically summarizes the latest mechanisms of immune escape in the renal cancer immune microenvironment, including abnormal expression of histocompatibility complex (MHC), secretion of immunosuppressive factors, programmed death ligand-1 with abnormal expression, recruiting immunosuppressive cells, and VHL gene deletion. This article also summarizes new treatment strategies proposed for these immune escape mechanisms. We hope this will help future researchers further explore the immune escape mechanism of renal cell carcinoma and propose new immunotherapy strategies.

## 1. Introduction

Renal cancer constitutes roughly 3% of all malignant neoplasms. The incidence rate of renal cancer ranks the third among urinary system tumors [1]. The annual occurrence rate of RCC (renal cell carcinoma) is increasing. Given the absence of distinctive early-stage symptoms, the majority of cases are detected during routine physical exams and are prone to being overlooked. When obvious symptoms such as hematuria, lower back pain, and abdominal masses appear, the condition has often progressed to the middle and late stages [2]. Renal cancer is insensitive to chemotherapy and radiotherapy, and the effectiveness of traditional treatment methods is very limited [3]. Late-stage patients rely on targeted therapy (such as sorafenib, sunitinib) or immunotherapy (such as PD-1 inhibitors) but are prone to developing drug resistance [4]. Renal cancer and immunity have a bidirectional regulatory relationship. Due to the immune monitoring function of the immune system, abnormal cells can be eliminated early to prevent cancer, while renal cancer weakens immune defense through immune escape mechanisms and can even promote tumor progression [5]. Despite extensive research on how kidney cancer can avoid immune damage, discovering effective immunotherapy remains a daunting task for researchers and clinical doctors. It is crucial to systematically study the immune escape mechanism of renal cell carcinoma to improve the effectiveness of immunotherapy and reduce drug resistance. The new targets for immunotherapy of kidney cancer need to be promoted simultaneously in both basic and clinical trials, and the combination therapy can bring more hope to kidney cancer patients. In this article, we systematically outline the immune escape mechanism of RCC, with a focus on the latest research progress and corresponding new treatment strategies.

## 2. Overview of the Immune Microenvironment in Renal Cancer

In the progression of tumors, the immune microenvironment of tumors cannot be ignored. The interactive environment formed by immune cells, stromal cells, and extracellular matrix during tumor progression is called the immune microenvironment [6,7]. The immune microenvironment plays an important role in inhibiting and killing tumor cells and promotes tumor development because tumor cells can evade the killing of immune cells [8]. The immune microenvironment of renal cell carcinoma is mainly composed and regulated by immune cells within the renal cell carcinoma tissue, including TAMs (Tumor-Associated Macrophages), NK cells (Natural Killer Cells), Tregs (Regulatory T Cells), DC cells (Dendritic Cells), other types of T cells and B cells [9,10,11]. The immune microenvironment in RCC can be broadly categorized into two distinct types: immune-activating and immune-suppressive [12]. The immunosuppressive type primarily enhances tumor progression by utilizing immune-suppressing cells and molecules, including M2 macrophages, Tregs, and TGFβ. Conversely, the immune-promoting type mainly exerts anti-tumor effects through immune-activated cells and pro-inflammatory cytokines [13,14].

When cancer occurs, the first step is to transform cells, disrupt immune balance, utilize the catalytic effect of inflammation, reshape CAFs (Cancer-Associated Fibroblasts) and ECM (Extracellular Matrix), and promote angiogenesis [15]. Immune cells in TME initially flood in to attempt to clear transformed cells. However, as the disease progresses, the immune environment gradually changes, activated T cells and myeloid cells accumulate, and genes involved in immune suppression are upregulated, leading to immune escape [16]. As the tumor progresses, cytotoxic CD8^+^ T cells and NK cells in TME decrease, while dysfunctional CD8^+^ T lymphocytes increase. Tumor cells secrete signaling molecules like VEGF to stimulate angiogenesis, supplying the tumor with oxygen and essential nutrients for growth [17]. The primary tumor forms a pre-metastatic niche through the interaction between paracrine and local stromal cells, immune cells, etc., promoting the spread of metastasis [18].

During the initial stages of RCC, tumor tissue exhibits infiltration by various immune cells, such as helper T cells, DC cells, NK cells, and cytotoxic T cells. Given the pronounced immunogenicity of early-stage RCC, these immune cells significantly contribute to immune surveillance and tumor cell destruction within the tumor microenvironment [19]. RCC cells are capable of releasing a range of cytokines and chemical messengers, IL-2, which aid in activating T cells and curbing tumor expansion [20]. Nevertheless, over time, tumor cells can evade immune system detection and destruction through genetic mutations or alterations, and may even manipulate immune cells to foster immune evasion and spur rapid growth [21]. At this juncture, immune evasion emerges as the predominant feature of the immune microenvironment in RCC. In comparison to other tumors, the immune microenvironment of renal cancer exhibits notable traits, such as an inverse relationship between CD8^+^ T cell infiltration and prognosis, an abundance of immunosuppressive macrophages, and a distinct hypoxic microenvironment that drives immune evasion [9]. Moreover, in renal cancer, immune escape is often the main reason for accelerated disease progression and immune therapy resistance in late-stage patients [22,23]. In summary, kidney cancer cells in the microenvironment of kidney cancer adopt various strategies to evade immune system attacks, leading to cancer progression, metastasis, resistance to immunotherapy, and susceptibility to drug resistance.

## 3. Abnormal Expression of Major Histocompatibility Complex 1

### 3.1. Function and Immune Recognition Mechanism of MHC Molecules

MHC molecules are critical for antigen presentation and are primarily classified into two types: MHC-I and MHC-II. MHC-I is expressed on the surface of all nucleated cells, where it presents endogenous antigens to CD8^+^ T cells, initiating their activation and subsequent tumor cell elimination [24]. MHC-II is predominantly found on antigen-presenting cells, where it displays exogenous antigens to CD4^+^ T cells, triggering the activation of adaptive immunity [25]. Immune escape is usually caused by downregulation of MHC-I expression. Due to the recognition of tumor cell antigens by CD8^+^ T, it is one of the main ways in which immune cells kill tumors; downregulation of MHC-I through tumor antigens prevents presentation to CD8^+^ T cells, leading to impaired recognition by CD8^+^ T cells and an inability to kill renal cancer cells [26,27]. In addition, MHC-I deficient renal cancer cells secrete chemokines (such as CCL2, CXL12) to recruit immunosuppressive cells, further inhibiting anti-tumor immune responses [28,29]. The reduction in MHC-I expression levels is also closely related to treatment resistance. According to reports, the decrease in MHC-I expression impairs the functionality of CD8^+^ T cells, ultimately leading to cancer’s resistance against immune checkpoint inhibitors [30,31,32].

### 3.2. Reduction in MHC-I Expression Levels

The expression process of proteins involves the transmission of genetic information from DNA to proteins, mainly divided into two core stages: transcription and translation, accompanied by post-translational modifications [33]. The immune evasion observed in RCC is strongly linked to the depletion of MHC-I protein, and the mechanism involved in MHC-I loss is very complex.

Firstly, abnormal regulation related to transcription leads to reduced expression of the MHC-I protein. For instance, in RCC, where there is a large infiltration of MARCO^+^ TAM, MARCO upregulates SOCS1 expression and inhibits JAK1 kinase activity, resulting in downregulation of MHC-I expression by suppressing the JAK1-STAT1-NLRC5 signaling cascade. Therefore, it inhibits MHC-I-mediated antigen cross-presentation to hinder CD8^+^ T development [26].

In addition, the transcribed MHC-I protein also undergoes antigen processing-related transporter transport and proteasome processing. If renal cancer cells downregulate the expression of proteasome subunits (such as LMP-2), it affects the generation and processing of antigen peptides, thereby reducing the formation of MHC-I antigen peptide complexes [34]. At the same time, there is also inhibition of antigen processing associated transporters (TAP), since TAP facilitates the transfer of antigenic peptides to the endoplasmic reticulum for MHC-I binding. Downregulation of TAP1 and TAP2 expression in renal cancer cells can block the transport of antigen peptides, resulting in MHC-I molecules being unable to load antigen peptides and being recognized and degraded by the endoplasmic reticulum-associated degradation pathway (ERAD) [35,36]. Liu Xing et al. found that overexpression of the immune response gene 1 (IRG1) elevates ROS levels by promoting the PPP and then promotes the expression of antigen processing-related transporters (TAP1, PSMB9) to increase MHC-I levels [36].

**Figure 1:**
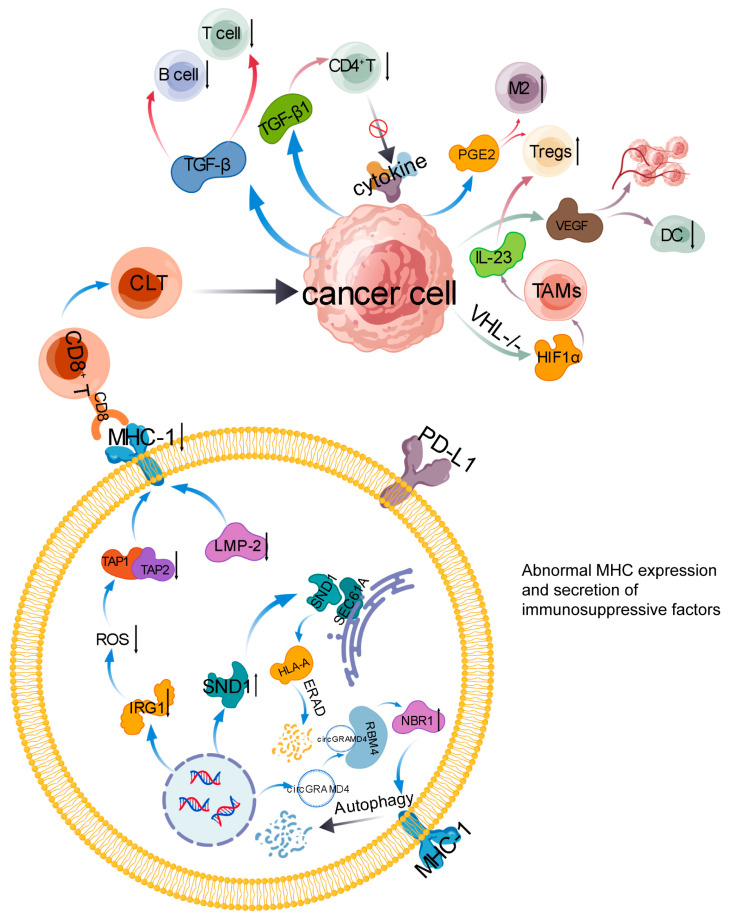
Abnormal expression of major histocompatibility complex 1 and secretion of immunosuppressive factors. TGF-β: Transforming Growth Factor-β, PEG2: Polyethylene Glycol 2, IL23: Interleukin-23, VHL: Von Hippel-Lindau Disease, HIF1α: Hypoxia-Inducible Factor-1α, VEGF: Vascular Endothelial Growth Factor, DC: Dendritic Cell, CTL: Cytotoxic T Lymphocyte, MHC-I: Major Histocompatibility Complex Class I, TAP1 and TAP2: Transporter Associated with Antigen Processing 1 and 2, ROS: Reactive Oxygen Species, IRG1: Immune-Responsive Gene 1, SND1: Staphylococcal Nuclease Domain-Containing Protein 1, HLA-A: Human Leukocyte Antigen-A, ERAD: Endoplasmic Reticulum-Associated Degradation, SEC61A: Sec61 Alpha Subunit, RBM4: RNA Binding Motif Protein 4, NBR1: Neighbor of BRCA1 Gene 1, circGARMD4: Circular RNA GARMD4, Tregs: Regulatory T Cells. The black up arrow indicates an increase, and the black down arrow indicates a decrease.

In addition, protein-level regulation is also crucial. There are reports that SND1 is significantly increased in ccRCC [37]. SND1 can be fixed on the endoplasmic reticulum membrane by binding to SEC61A, promoting the degradation of immature MHC-I molecules (such as HLA-A) through the ERAD pathway, thereby reducing the MHC-I protein on the cell membrane. SND1 deficiency can markedly reinstate MHC-I expression, boost CD8^+^ T cell infiltration and enhance its ability to kill tumor cells [38,39]. Some have reported that renal cancer cells can break down MHC-I molecules via the ubiquitin-proteasome pathway or autophagy pathway. Circular RNA circGRAMD4 is significantly elevated in renal cell carcinoma and interacts with RBM4 protein, stabilizing autophagy cargo receptor NBR1 mRNA to promote NBR1 expression. This results in the breakdown of MHC-I molecules via major autophagy pathways, affecting antigen presentation in RCC cells and ultimately inducing CD8^+^ T cell dysfunction, which contributes to tumor immune escape [40]. (This process is shown in Figure 1.)

## 4. Secretion of Immunosuppressive Factors

During the immune escape process of renal cell carcinoma, renal cell carcinoma cells secrete multiple immunosuppressive agents, including transforming growth factor-beta (TGF-β), PGE2 (prostaglandin E2), VEGF (vascular endothelial growth factor) and IL-23 (interleukin-23) are immunosuppressive factors that hinder immune cell activation and proliferation, thereby weakening the immune system’s anti-tumor capacity [41].

There are reports that TGF-β can inhibit T cell proliferation and IL-2 receptor expression, weaken B cell function, and promote tumor angiogenesis [42]. Moreover, high TGF-β levels in RCC are closely linked to tumor progression and immune escape, as this cytokine inhibits CD8^+^ T cell function, allowing sustained tumor growth in vivo [43,44]. F Cottrez et al. found that TGF-β1 is an important pleiotropic cytokine that stimulates and inhibits cell growth and differentiation. TGF-β1 inhibits the proliferation of CD4^+^ T cells and induces their differentiation into Tregs through the classical SMAD signaling pathway (such as SMAD2/3), promoting immune escape of tumor cells [45].

Furthermore, research indicates that pro-inflammatory prostaglandin E2 (PGE2) significantly contributes to cancer development and progression by activating its corresponding EP receptors, which are part of the G protein-coupled receptor superfamily [46]. In addition, PGE2 can prompt the formation of suppressive T cells, diminish the activity of LAK cells, and hinder T cell proliferation. Additionally, by orchestrating and curbing the production of type I interferon cytokines, it prevents excessive MAPK signaling activation in cancer cells, consequently weakening the inflammatory monocyte phenotype and suppressing T cell activation within the tumor [47].

Meanwhile, VEGF protein in tumor cells can cause neovascularization, inhibit dendritic cell maturation, and weaken antigen presentation ability [48]. Some studies indicate that PinX1’s transcriptional activation of miR-125a-3p expression suppresses the VEGF, a target gene of miR-125a-3p, thus facilitating DC cell maturation and curbing immune evasion in renal cancer. Consequently, miR-125a-3p has been suggested as a promising novel therapeutic target for RCC, specifically targeting VEGF [49].

Some people have reported that renal cancer cells can consume glutamine, leading to a decrease in extracellular glutamine, thereby inducing tumor infiltrating macrophages to secrete IL-23 by activating hypoxia inducible factor 1 alpha. IL-23 drives Tregs expansion and enhances TGF-β/IL-10 production, which suppresses CD8^+^ T cell and NK cell-mediated tumor cell killing, thereby fostering immune evasion in renal cancer [50]. (This process is shown in Figure 1.)

## 5. Upregulation of Programmed Death Ligand-1

PD-L1 acts as a key mechanism enabling tumor cells to evade immune detection and destruction [51]. PD-L1 expression serves as a crucial biomarker for forecasting the effectiveness of immune checkpoint inhibitors. When tumor cells exhibit high PD-L1 levels on their surface, it binds to PD-1 on T cells, suppressing T cell function and rendering them ineffective at killing tumor cells, ultimately enabling tumor cell immune evasion [52].

Similarly to MHC-I, the expression regulation process of PD-L1 conforms to the general protein synthesis regulation process, such as transcriptional upregulation. It has been reported that ccRCC exhibits enhanced glycolysis. As the glycolysis-related gene (GRG) HK3 is a key regulatory factor for O-GlcNA acylation in ccRCC, O-GlcNA acylation is located at the Ser900 site of EP300. Therefore, its stability and carcinogenic activity can be enhanced by preventing ubiquitination. EP300, which is stably expressed, acts as a co-transcription factor with TFAP2A to promote PD-L1 transcription, and acts as an acetyltransferase to stabilize PD-L1 protein and promote tumor cells to evade T cell-mediated immune surveillance [53]. Y Adachi et al. found that the activation of fibroblast growth factor receptor signaling suppresses the JAK/STAT signaling pathway activated by IFN-γ. This regulation of transcription factors reduces the expression of their target gene (PD-L1) and can also facilitate renal cancer cells in evading T cell immune surveillance [54].

In addition, recruiting promoters is also an important way to regulate PD-L1 expression. Compared with normal tissues, there was an overall change in the inclusion rate of polybroma 1 (PBRM1). One of the subunits of the PBAF SWI/SNF chromatin remodeling complex is PBRM1, specifically exon 27 (E27) in RCC tissues. The direct binding of RBFOX2 to the intron UGCAUG element excludes PBRM1 E27. PBRM1 subtype containing E27 can enhance the recruitment of PD-L1 promoter by PBAF complex to upregulate PD-L1 expression [55].

Meanwhile, inflammation signaling and epigenetic changes significantly influence PD-L1 expression. Studies indicate that TET2 can maintain IRF1 demethylation and promote PD-L1 expression [56]. Liu Wentao et al. also found that the dysregulation of activated T cell nuclear factor 1 (NFAT1) leads to the progression of diverse malignant tumors, particularly RCC, and is notably impacted by aberrant overexpression, which can dampen T cell immune responses and foster tumor growth through elevated PD-L1 expression in RCC [57].

**Figure 2:**
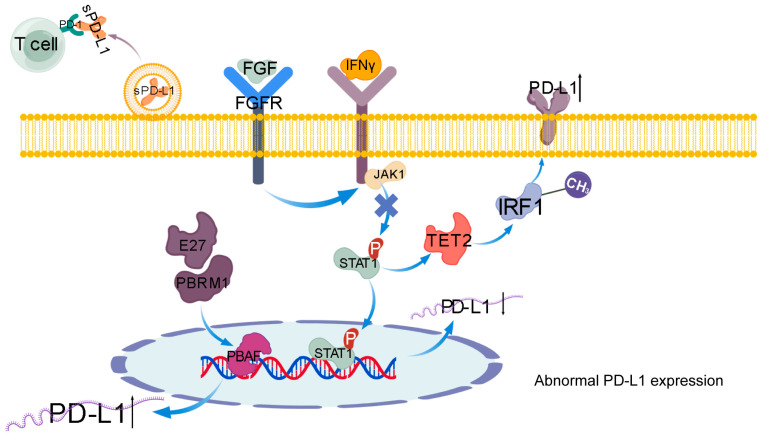
Upregulation of PD-L1. sPD-L1: Soluble Programmed Death-Ligand 1, FGFR: Fibroblast Growth Factor Receptor, IFNγ: Interferon-gamma, PBRM1: Polybromo-1, JAK1: Janus Kinase 1, STAT1: Signal Transducer and Activator of Transcription 1, TET2: Ten-Eleven Translocation 2, IRF1: Interferon Regulatory Factor 1, FGF: Fibroblast Growth Factor and FGFR: Fibroblast Growth Factor Receptor, PBRM1: Polybromo 1, PBAF: Polybromo-associated BAF, IFNγ: Interferon-gamma. The black up arrow indicates an increase, and the black down arrow indicates a decrease.

Furthermore, renal cancer cells not only evade immune surveillance through membrane surface PD-L1 but also secrete soluble PD-L1 (sPD-L1) through exosomes [58]. SPD-L1 can both locally act on tumor infiltrating T cells and be widely distributed through blood circulation, triggering systemic immune suppression [59]. The concentration of sPD-L1 in cancer patients’ blood shows a positive association with tumor severity and inhibits the therapeutic effect of immune checkpoint inhibitors [60]. (This process is shown in Figure 2.)

## 6. Recruit Immunosuppressive Cells

In advanced renal cancer, a multitude of immunosuppressive cells—including regulatory T cells, MDSCs, TAMs, and PMN-MDSCs—congregate in TME, inhibiting immune-mediated tumor destruction and promoting renal cancer immune escape.

### 6.1. Regulatory T Cells and Immune Escape

The characteristic of ccRCC is the immunosuppressive tumor microenvironment, mainly composed of regulatory T cells, which is conducive to immune evasion [61]. Renal cancer cells can affect Tregs by secreting cytokines, such as the chemokine CCL22, which can recruit Tregs into tumor tissue. Tregs inhibit CD8^+^ T cell activity by expressing CTLA-4 and PD-1 while releasing IL-10 and TGF-β, culminating in a broad immunosuppressive response [61,62].

There are also reports that TGFBI is related to Treg regulation. TGFBI is a protein induced by TGF-β that promotes the secretion of CCL22 and neutralizes CCL22 to reverse Tregs chemotaxis. TGFβI recruits regulatory T cells through CCL22 to promote immune suppression of ccRCC, thereby promoting tumor progression and treatment resistance [61]. In addition, someone has discovered that knocking down the ubiquitin-binding UBE2-related gene (UBE2RG) in cells reduces the release of cytokine TGF-β1, leading to a decrease in the proportion of Tregs [63]. At the same time, renal cell carcinoma overexpresses certain immune-related genes, such as the immune-related gene VSIG4, which is notably overexpressed in ccRCC and functions similarly to other immune checkpoint ligands. Expressed prominently on antigen-presenting cells and ccRCC cells, it drives immune evasion by dampening T cell activity [64]. Cui Yankang et al. found that KCNN4 (an intermediate conductance calcium-activated potassium channel protein) is positively correlated with regulatory T cells. The high expression of KCNN4 may lead to immune suppression and inhibit the therapeutic effect of immune checkpoint inhibitors [65].

In addition, genes related to metabolism are also involved in Tregs regulation in renal cancer; for instance, glycerol-3-phosphate dehydrogenase 1-like (GPD1L), which is a tumor suppressor gene in ccRCC, and GPD1L are significantly reduced in ccRCC patients. The downregulation of GPD1L promotes tumor cell survival by enhancing the infiltration of Tregs in ccRCC and affecting lipid metabolism to form an immunosuppressive microenvironment [66].

### 6.2. Tumor Associated Macrophages and Immune Escape

TAMs include two types, namely M1 macrophages and M2 macrophages. M1 macrophages exhibit antitumor effects, while M2 macrophages support tumor progression. RCC is often infiltrated by TAMs and, overall, it is mainly tumor-promoting, thereby promoting malignant progression [67]. By secreting chemokines like CCL2, CCL17, and CCL22, tumor cells guide monocytes to the tumor site and drive their transformation into TAMs [68]. By releasing IL-10 and TGF-β, TAMs inhibit T cell and NK cell responses, while also directly blocking T cell activation through PD-L1 expression [69].

Furthermore, TAMs can promote tumor angiogenesis and invasion and metastasis [70]. Transcriptional regulation is crucial in the M2 polarization regulation of TAMs; among these are the CCAAT/enhancer binding proteins (CEBPs), essential transcription factors that govern macrophage pathological differentiation. CEBPD is a key transcription factor for TAM polarization in ccRCC. Through the RGS2/PAR2 signaling axis, CEBPD disrupts M1-like polarization in experimental and physiological settings, contributing to immune suppression [71].

In addition, TCF7L2 acts as a key transcription factor influencing the transcriptional diversity and differentiation of tumor-associated macrophages. It is notably overexpressed in TAMs and drives macrophage polarization towards the M2 phenotype. Elevated TCF7L2 levels in macrophages substantially boost the invasion and proliferation of ccRCC. Moreover, TCF7L2 facilitates immune evasion in ccRCC by promoting TAM recruitment and enhancing their M2 polarization [72]. Increased levels of BASP1 have been linked to immune suppression, reduced responsiveness to immune checkpoint inhibitors, and abnormal macrophage buildup, according to recent findings. In RCC, in macrophages, especially M2 macrophages, BASP1 elevates EGFR expression, which in turn enhances activation of the PI3K/AKT signaling pathway. This, in turn, drives the polarization of macrophages towards the M2 phenotype and fosters tumor progression [73].

Meanwhile, secretory phosphoprotein 1 (SPP1)-positive tumor-associated macrophages display immunosuppressive and tumor-promoting characteristics. They are linked to compromised effector function and the terminal differentiation of CD8^+^ T cells, resulting in a suboptimal response to immunotherapy in patients with high infiltration of SPP1^+^ TAMs [74].

Metabolic regulation cannot be ignored in the immune escape mechanism of RCC. TAMs within renal cell carcinoma stimulate eicosanoid generation, particularly CCL2 and IL-10, via activation of the 15-lipoxygenase 2 (15-LOX2) signaling cascade. By upregulating FOXP3 and CTLA-4 in T cells without lipoxygenase involvement, TAMs drive immune suppression and malignant advancement in RCC [67].

### 6.3. Myeloid Suppressor Cells and Immune Escape

MDSCs mediate severe immune suppression. This diverse group of immature bone marrow cells has the capacity to significantly suppress the anti-tumor functions of NK and T cells, while also promoting the activation of Tregs, form an immunosuppressive microenvironment, and lead to tumor progression [75]. There are reports that renal cell carcinoma-derived extracellular vesicles (RDEs) can present antigen information, and activate and drive specific immune suppression of MDSCs. Through TLR2-mediated signaling driven by high HSP70 levels in RDEs, MDSCs induced by RDEs produce antigen-specific immunosuppression of cytotoxic T cells, specifically aiding renal tumor growth and immune evasion [76]. Research has also indicated that papillary renal cell carcinoma (pRCC) frequently exhibits the loss of tumor suppressor factors upstream of the Hippo/YAP signaling pathway. In pRCC, hyperactivated YAP1 triggers epithelial cell transformation and promotes the accumulation of MDSCs by modulating multiple signaling pathways. Reducing MDSC levels can inhibit YAP1-induced renal overgrowth and tumorigenesis [77]. In addition, POP7 mRNA and protein are highly expressed in ccRCC. POP7 is positively correlated with Tregs and MDSCs in renal cancer, and is an immunosuppressant of ccRCC, promoting renal cell carcinoma immune escape [78].

Li Junyi et al. found that elevated TUBA1C expression activates the PI3K/AKT pathway, boosting the infiltration of Tregs and MDSCs, thereby fostering an immunosuppressive microenvironment in ccRCC [79]. Research has revealed that certain medications can trigger the recruitment of myeloid-derived inhibitory cells. As an FDA-approved treatment for pulmonary and renal fibrosis and an effective TGF-β blocker, pirfenidone (PFD) reduces TGF-β levels upon use. This, in turn, restricts the recruitment of tumor-infiltrating MDSCs and modifies the immunosuppressive tumor microenvironment [80].

**Figure 3:**
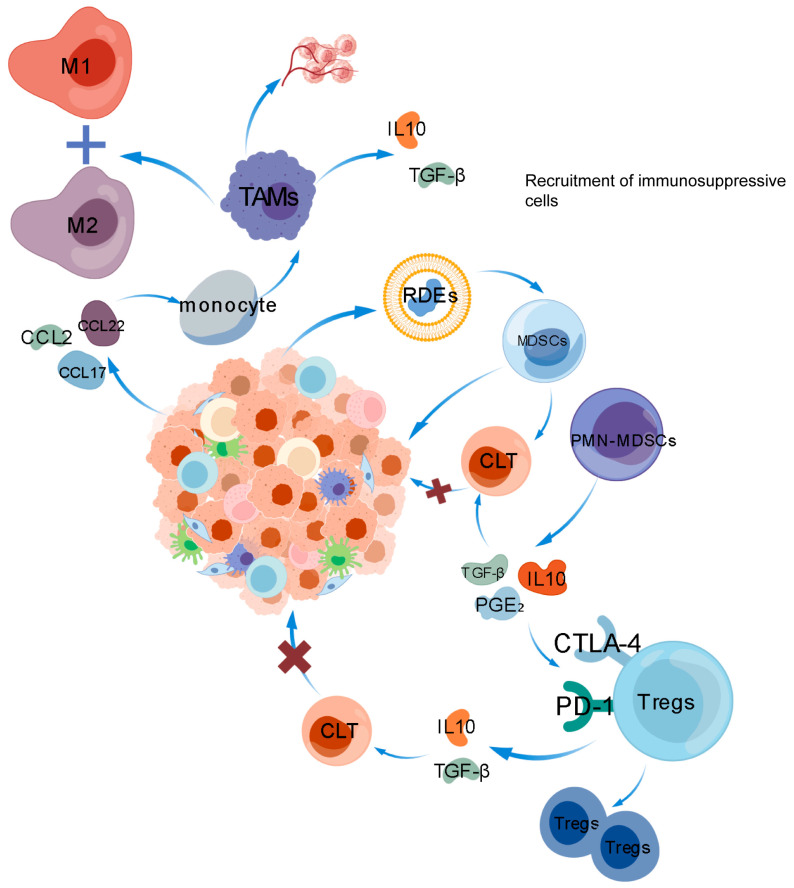
Recruit immunosuppressive cells. IL10: Interleukin-10, TAMs: Tumor-Associated Macrophages, CCL2 and CCL17 and CCL22: Chemokine (C-C Motif) Ligand 2, 17, 22, RDEs: RNA-DNA Hybrids, MDSCs: Myeloid-Derived Suppressor Cells, PMN-MDSCs: Polymorphonuclear Myeloid-Derived Suppressor Cells, PD-1: Programmed Cell Death Protein 1, CTLA-4: Cytotoxic T-Lymphocyte-Associated Protein 4, Tregs: Regulatory T Cells. Red ✕ indicates blocking. The blue arrow indicates the next step.

PMN-MDSCs are also crucial in immune escape in renal cancer. PMN-MDSCs are a major subset of MDSCs, belonging to granulocytic/PMN-MDSCs. PMN-MDSCs have a different immune suppression mechanism from MDSCs, in which PMN-MDSCs primarily utilize ROS, peroxynitrite, ARG1, and PGE2 to mediate immune suppression, and induce the expansion of Treg by secreting inhibitory cytokines such as IL-10, PGE_2_, and TGF-β and block antigen presentation, interfere with the expression of co-stimulatory molecules and antigen cross-presentation of dendritic cells (DCs), and comprehensively weaken tumor-specific immune responses [81,82]. In the process of immune escape in RCC, PMN-MDSCs recruited into the tumor can inhibit T cell activity and promote Tregs expansion through the production of reactive oxygen species, arginase, and other methods, further exacerbating the phenomenon of immune escape [83,84]. There are reports that the key molecule C3 in extracellular vesicles (EVs) derived from renal cancer cells can promote the secretion of chemokines CCL2 and CXCL1 by macrophages at the site of lung metastasis. These two chemokines mainly recruit PMN MDSCs, thereby promoting immune escape in renal cancer [28]. In addition, the cytokine IL1 β and its downstream mediators can promote the infiltration of PMN-MDSCs into tumors and angiogenesis, thereby producing immunosuppressive effects and promoting tumor immune escape [85]. (This process is shown in Figure 3.)

Therefore, renal cancer cells play an important role in the immune escape process of renal cancer by interacting with other tissue cells and recruiting various immunosuppressive cells.

## 7. Special Mechanism of VHL Gene Deletion

The hypoxic conditions within the primary tumor site of renal cell carcinoma significantly contribute to its invasion and metastasis [86]. Loss of VHL gene function in renal cancer leads to pseudo hypoxia, which promotes the upregulation of HIF-1 α/HIF-2α expression, activates and enhances downstream oncogene expression, which is also one of the important mechanisms of immune escape in RCC [87,88]. The hypoxic setting can spur tumor cells to secrete a range of immunosuppressive factors, thereby further hampering immune system function [89].

The characteristics of ccRCC are imbalance of hypoxic signaling and a highly enriched tumor microenvironment (TME) of bone marrow and lymphocytes [90]. The loss of the VHL gene represents a crucial early event in the development of ccRCC. Due to the promotion of HIF stability by VHL deletion, T cells living in a pseudo hypoxic environment have reduced function and response to PD-1 therapy [91]. Meanwhile, due to the stable immune suppression pathway driven by HIF, tumors with VHL mutations are less infiltrated by natural killer cells. VHL mutations can reshape the tumor microenvironment, accelerating the progression of ccRCC and enhancing immunosuppressive signals, while simultaneously suppressing the infiltration and activation of NK cells [92]. VHL loss is prevalent in ccRCC tumors, triggering HIF-α accumulation and transcriptional upregulation of hypoxia-responsive genes, including the VEGFA gene that encodes VEGF-A [93].

**Figure 4:**
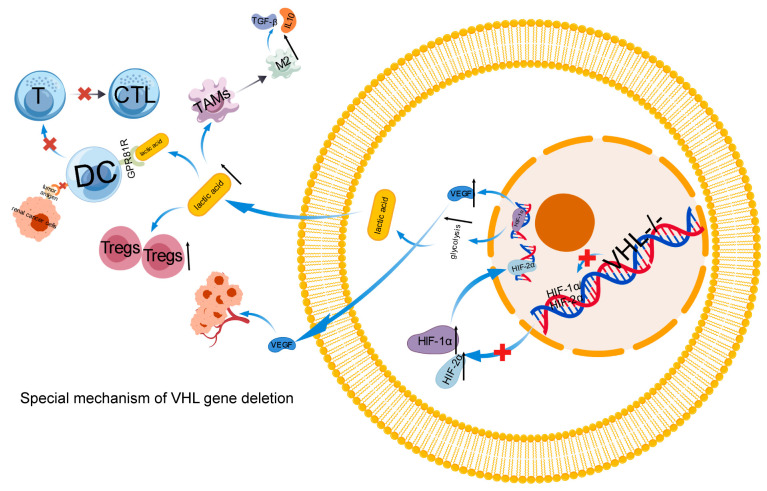
Special mechanism of VHL gene deletion. Tregs: Regulatory T Cells, VEGF: Vascular Endothelial Growth Factor, HIF-1αand HIF-2α: Hypoxia Inducible Factor 1 Alpha and Hypoxia Inducible Factor 2 Alpha, GPR18R: N-arachidonyl glycine receptor, TGF-β:Transforming Growth Factor Beta. The black up arrow indicates an increase, and the black down arrow indicates a decrease. Red ✕ indicates blocking. The blue arrow indicates the next step.

VEGF-A serves as a pivotal factor not only in tumor angiogenesis but also in exerting immunosuppressive effects by directly influencing CD8^+^ T cells within the tumor microenvironment. Antigen stimulation can induce VEGFR2 expression, allowing tumor-infiltrating CD8^+^ T cells to respond to VEGF-A. The interaction of VEGF-A with VEGFR2 boosts TOX expression, which governs CD8^+^ T cell exhaustion and results in T cell dysfunction [94,95,96]. (This process is shown in Figure 4.)

In summary, the immune microenvironment of RCC is extremely complex, and the immune escape of RCC also involves multiple mechanisms, but there are still many mechanisms that have not been studied and elucidated. Therefore, in the future, it will be necessary to continue exploring the immune escape mechanism of RCC while searching for more therapeutic targets for different mechanisms and proposing more effective treatment plans.

## 8. Treatment Strategies for Immune Escape

The immune escape mechanism of kidney cancer allows cancer cells to evade the attack of the immune system, and continue to proliferate and spread in the body, leading to tumor progression. Immune-escaping tumor cells are also more likely to metastasize to other areas through the blood or lymphatic system, forming new tumor lesions and increasing the risk of death for patients. Immune escape also affects the effectiveness of immunotherapy, making patients insensitive to immunotherapy and prone to developing drug resistance. Therefore, treatment methods designed for immune escape are crucial. At present, with the continuous deepening of research on the immune escape mechanism in RCC, many new treatment methods have been proposed. The following will discuss four aspects: MHC-I expression regulation, PD-L1 expression regulation, immune suppressive cell recruitment, and VHL gene deletion.

### 8.1. Immunotherapy Strategies for MHC-I

In renal cancer, the immune escape mechanism underlying changes in MHC-I expression is highly complex, and the focus of treatment strategies is on how to restore MHC-I expression. Firstly, the latest research is about the role of epigenetic modulators. Some studies have shown that epigenetic modulators can reverse the silencing of MHC genes and restore their expression [97,98], for instance, DNA methyltransferase inhibitors like 5-azacytidine and histone deacetylase inhibitors such as vorinostat [99].

In addition, the exploration of new targets is constantly advancing. As previously introduced, small molecule inhibitors targeting SND1 protein have been developed to block its degradation of MHC-I, thereby inhibiting immune escape in renal cancer [38,39]. As previously discussed, the immune response gene 1 (IRG1) promotes the expression of antigen processing-related transporters (TAP1, PSMB9) through the pentose phosphate pathway (PPP), thereby increasing MHC I levels. Therefore, the NADPH oxidase (NOX) inhibitor DPI has been developed for the pentose phosphate pathway, which can reduce TAP1 and MHC-I levels in macrophages overexpressing IRG1. Therefore, the development of agonists for NADPH oxidase (NOX) is crucial [36]. This research on metabolic regulation and immune escape has also received much attention, and there are reports that exogenous supplementation of alpha ketoglutarate (alpha KG) can promote MHC-I antigen processing and presentation in renal cancer cells, as well as the expression of β 2-microglobulin (B2M) to eliminate the immunosuppressive characteristics of renal cancer [31].

In addition, studies have found that the novel histone deacetylase (HDAC) inhibitor OBP-801 can upregulate MHC-I by inducing the expression of low molecular weight peptide (LMP) 2 in renal cancer cells [34].

In renal cancer, beyond reinstating MHC-I expression, there are also pathways that do not rely on MHC-I molecules to activate immune anti-tumor therapy and develop new treatment methods based on this. If T cells are genetically engineered to express TCR capable of recognizing kidney cancer-associated antigens, bypassing MHC-I restriction recognition. There have been reports of screening for oncogenic KRAS G12V mutation conjugates targeting peptide MHC complexes and integrating new antigen conjugates into CAR-T cells (mKRAS NeoCAR), demonstrating their effectiveness in renal cell carcinoma xenograft models [100]. There are also ways to modify NK cells by utilizing their natural killing activity against MHC-I deficient cells, thereby enhancing their clearance of renal cancer cells [101].

In addition, combination therapy has always been an important means of immunotherapy. If PD-1/PD-L1 antibodies and epigenetic regulators are used in combination, they can simultaneously enhance T cell activity and restore MHC expression, thereby improving anti-tumor efficacy [34,102]. In addition, combining immune checkpoint inhibitors with targeted SND1 drugs can not only block SND1 function and restore MHC-I expression but also enhance the efficacy of immune checkpoint inhibitors, thereby improving the tumor killing effect [38,39].

### 8.2. Immunotherapy Strategies Targeting PD-L1

The immunotherapy strategy of PD-L1 is to block the interaction between PD-L1 and PD-1, restore the anti-tumor activity of T cells, and thus inhibit tumor growth and spread [103]. Therefore, research on targeted therapies developed solely for PD-L1 is in full swing. If someone discovers that via the IFN-γ-STAT1 signaling axis, TET2 binds to IRF1, sustaining its demethylation and subsequently upregulating PD-L1. This pathway promotes T cell infiltration and the production of cytokines and chemokines. Additionally, supplementing with ascorbic acid (vitamin C) can synergistically boost TET activity, thereby improving immunotherapy for RCC. Therefore, ascorbic acid enhances T cell infiltration and cytokine/chemokine expression in tumors, leading to heightened immune cell-mediated tumor destruction [56]. At present, many new targets have been developed for PD-L1, such as RRM2 [104] and NFAT1 [57].

Moreover, the combination of anti-PD-1 therapy with other drugs in immunotherapy is currently a research hotspot, and discovering more effective combination methods is key. A study revealed that combining anti-PD-1 monoclonal antibodies with VEGFR-selective inhibitors like lenvatinib or axitinib boosts anti-tumor effects in RCC. However, fibroblast growth factor receptor-signaling activation suppresses the IFN-γ-stimulated JAK/STAT pathway, leading to reduced PD-L1 expression. Notably, lenvatinib decreased tumor-associated macrophages while increasing CD8^+^ T cell numbers [54]. In addition, approximately 70% of renal cancer cases are ccRCC with activation of the VEGF pathway. Therefore, tyrosine kinase inhibitors targeting VEGF have become important drugs for the treatment of ccRCC. Notably, combining PD-1/PD-L1 blockade with TKI therapy improves overall survival in RCC patients [105].

### 8.3. Immunotherapy Strategies Targeting Immunosuppressive Cells

Various immune cells are pivotal in facilitating immune evasion in renal cell carcinoma. We will focus on introducing immunotherapy strategies targeting regulatory T cells, MDSCs, and PMN-MDSCs.

#### 8.3.1. Regulatory T Cells

At present, many new targets have been proposed for regulatory T cells. A study has found that in RCC, due to the important role of CXCR4 in regulating T cell transport, the novel CXCR4 antagonist R54 can inhibit regulatory T cell activity in primary RCC patients by lowering TSDR demethylation along with decreasing FOXP3 and DNMT1 expression. Therefore, focusing on CXCR4 represents an innovative immunotherapy approach for RCC [106]. Additionally, the research revealed that the immune-related gene VSIG4 facilitated Tregs infiltration in ccRCC tissues. By showing high expression on antigen-presenting cells and ccRCC cells, it suppresses T cell activity and supports immune evasion, highlighting VSIG4 as a promising new immunotherapeutic target for ccRCC patients unresponsive to existing therapies [64]. Meanwhile, targeting the TGFBI-CCL22 axis can increase the potency of immunotherapy and suppress immune escape of ccRCC by inhibiting Tregs [61]. There are also studies on the involvement of metabolites in immune escape of RCC, which have the potential to become important treatment strategies. Regulating LOX-dependent arachidonic acid metabolism in TME can inhibit regulatory T cell activity to block immune escape in renal cell carcinoma patients [67].

#### 8.3.2. Myeloid Derived Inhibitory Cells and PMN Myeloid Derived Inhibitory Cells

At present, research has discovered a new direction for inhibiting immune escape in RCC, which is to target and eliminate the function of MDSCs, or eliminate the expression of HSP70 in extracellular vesicles, or block their crosstalk [76]. If MGH-CP1 (a recently developed TEAD inhibitor) is used to inhibit YAP1 activity, it can hinder MDSC accumulation and inhibit tumor development [77]. Moreover, TUBA1C expression triggers the PI3K/AKT pathway, leading to an enhanced infiltration of bone marrow-derived suppressor cells. It is expected to target TUBA1C as a therapeutic target to enhance responsiveness to ICB [79].

In addition, as angiogenic factors and their receptors can directly act on immune cells and affect endothelial cells to promote the formation of an immunosuppressive microenvironment. Therefore, combining immune checkpoint inhibitors with VEGFR tyrosine kinase inhibitors has emerged as a new standard first-line treatment for patients with advanced RCC. For instance, targeted anti-angiogenic therapy can boost the infiltration of mature DC cells and effector T cells into tumors, while decreasing the presence of Tregs and MDSCs, thus inhibiting immune escape [107].

There are also reports that modulation of MDSCs through immune cell modification, for instance, the simultaneous delivery of IL-12 and CXCL-9 via macrophages engineered with dual cytokines can reshape the TME and improve the response to anti-PD-1 therapy. The aggregation of engineered macrophages in tumors through intravenous injection can increase CD8^+^ T cells, DCs, and NKs to reshape TME, while reducing immune suppressive Tregs [108].

Given their strong influence on immunotherapy effectiveness, MDSCs are crucial for informing personalized treatment strategies. Determining the baseline levels of bone marrow-derived suppressor cells, particularly the monocytes and PMN-MDSC subtypes, can predict the response to ICIs in patients with ccRCC. The initial concentration of circulating M-MDSCs and their proportion among MDSCs might function as biomarkers for immune checkpoint inhibitor outcomes in ccRCC [109]. The successful treatment inhibition of PMN-MDSCs and the effective synergy with ICB have been studied, which can eradicate refractory malignant tumors of the urogenital system caused by ICB [84].

### 8.4. Immunotherapy Strategies for VHL

The deletion of the VHL gene occurs early in RCC development and can be identified in both early and advanced stages, showing no notable difference in incidence rates [110]. In VHL-deficient tumors, tumor-associated macrophages (TAMs) exhibit enhanced glucose consumption and phagocytosis, while lymphocytes show reduced activation levels and decreased responsiveness to in vivo anti-PD-1 therapy [91]. Although VHL deficiency leads to increased immune cell infiltration, decreased lymphocyte activation levels and reduced responsiveness to immune checkpoint blockade therapy limit the effectiveness of immunotherapy in VHL-deficient RCC [21,111]. Therefore, how to improve the responsiveness of immunotherapy in VHL-deficient RCC is an important direction of current research. Due to the inhibition of HIF-α in RCC, it can promote the infiltration of natural killer cells into VHL mutant tumors [92]. Therefore, inhibition of HIF α should be explored as a therapeutic strategy for ccRCC to enhance the anti-tumor efficacy of natural killer cells against VHL mutant tumors. This chapter is summarized in Table 1.

## 9. Conclusions

With more research revealing the mechanism of immune escape in RCC, this will benefit more renal cell carcinoma patients. This article summarizes the anti-tumor effect of renal cancer cells by suppressing immune cells through five mechanisms: altered MHC-I expression and the release of immunosuppressive factors, expression of PD-L1, recruitment of immunosuppressive cells, and VHL gene deletion. It also discusses new therapeutic strategies proposed for immune escape targeting these mechanisms. In summary, to achieve successful treatment of renal cancer, it is essential to take into account both the renal cancer cells and the immune cells present within the tumor. This is mainly reflected in the need to suppress the immunosuppressive properties of renal cancer cells themselves while enhancing the anti-tumor effect of immune cells. As research on the mechanism of interaction between RCC and immunity becomes progressively more thorough, and treatment plans advance through interdisciplinary collaboration, continuous research in clinical technology and basic studies, immunotherapy for renal cell carcinoma will make greater progress.

## Figures and Tables

**Table 1:** This table summarizes treatment strategies and immune escape choices.

Restore the Expression of MHC-I	Block the Interaction Between PD-L1 and PD-1	Target Immune Suppressive Cells and VHL Gene Deficiency
Epigenetic regulators: DNA methyltransferase inhibitor (5-azacytidine) and histone deacetylase inhibitor (vorinostat)	Targeted development for PD-L1	Regulatory T cells: A novel CXCR4 antagonist R54 targets VSIG4 and TGFβ1-CCL22
SND1 inhibitor, NADPH oxidase (NOX) inhibitor DPI, Exciters of NADPH oxidase (NOX), Exogenous supplementation of alpha ketoglutaric acid (alpha KG)	Ascorbic acid treatment	Myeloid-derived inhibitory cells and PMN myeloid-derived inhibitory cells: MGH-CP1 (a recently developed TEAD inhibitor), targeting TUBA1C,
New histone deacetylase (HDAC) inhibitor OBP-801, Bypass MHC-I restriction recognition: modify NK cells, CAR-T cells (mKRAS NeoCAR)	New targets: RRM2 and NFAT1	co-delivering IL-12 and CXCL-9 by dual cytokine engineered macrophages
Combination use of PD-1/PD-L1 antibodies and epigenetic regulators, Combination of immune checkpoint inhibitors and targeted SND1 drugs	Combination therapy of anti-PD-1 mAb and VEGFR selective inhibitors lenvatinib or axitinib	VHL gene deletion: inhibits HIF α and natural killer cell infiltration

## Data Availability

No new data were created or analyzed in this study. Data sharing is not applicable to this article.

## Abbreviations

The following abbreviations are used in this manuscript:

CCL17	Chemokine (C-C motif) Ligand 17
CCL2	Chemokine (C-C motif) Ligand 2
CCL22	Chemokine (C-C motif) Ligand 22
CEBPs	CCAAT/Enhancer Binding Proteins
CircGRAMD4	Circular RNA GRAMD4
CTLA-4	Cytotoxic T-Lymphocyte Antigen 4
CXCL12	Chemokine (C-X-C motif) Ligand 12
CXCR4	C-X-C Chemokine Receptor Type 4
DC	Dendritic Cell
EP300	E1A Binding Protein P300
ERAD	Endoplasmic Reticulum-Associated Degradation
EVs	Extracellular Vesicles
FOXP3	Forkhead Box P3
GPD1L	Glycerol-3-Phosphate Dehydrogenase 1-Like
HIF-1 α/HIF-2	Hypoxia-Inducible Factor 1 Alpha/Hypoxia-Inducible Factor 2 Alpha
HLA-A	Human Leukocyte Antigen-A
HDAC	Histone Deacetylase
HSP70	Heat Shock Protein 70
ICB	Immune Checkpoint Blockade
IFN-γ	Interferon Gamma
IL-10	Interleukin-10
IL-2	Interleukin-2
IL-23	interleukin-23
IRF1	Interferon Regulatory Factor 1
IRG1	Immune-Responsive Gene 1
JAK1	Janus Kinase 1
KCNN4	Potassium Calcium-Activated Channel Subfamily N Member 4
LMP	Low Molecular Weight Protein
LMP-2	Low Molecular Weight Protein 2
MDSCs	Myeloid-Derived Suppressor Cells
MHC	Major Histocompatibility Complex
MHC-I	Major Histocompatibility Complex Class I
MHC-II	Major Histocompatibility Complex Class II
NLRC5	NLR Family CARD Domain Containing 5
NFAT1	Nuclear Factor of Activated T-cells 1
NK	Natural Killer Cell
O-GlcNA	O-linked N-acetylglucosamine
PBRM1	Polybromo-1
PBAF SWI/SNF	Polybromo-associated BAF SWI/SNF
PD-1	Programmed Death-1
PD-L1	Programmed Death-Ligand 1
PGE2	Prostaglandin E2
PI3K/AKT	Phosphatidylinositol 3-Kinase/Protein Kinase B
PMN-MDSCs	Polymorphonuclear Myeloid-Derived Suppressor Cells
POP7	Processing Of Precursor 7
PFD	Pre-foldin
PSMB9	Proteasome Subunit Beta Type-9
RBFOX2	RNA Binding Fox-1 Homolog 2
RCC	Renal Cell Carcinoma
RGS2/PAR2	Regulator of G Protein Signaling 2/Protease-Activated Receptor 2
RRM2	Ribonucleotide Reductase Regulatory Subunit M2
sPD-L1	Soluble Programmed Death-Ligand 1
SOCS1	Suppressor of Cytokine Signaling 1
SND1	Staphylococcal Nuclease Domain-Containing 1
SPP1	Secreted Phosphoprotein 1
STAT1	Signal Transducer and Activator of Transcription 1
TAMs	Tumor-Associated Macrophages
TAP1	Transporter Associated with Antigen Processing 1
TAP2	Transporter Associated with Antigen Processing 2
TCF7L2	Transcription Factor 7-Like 2
TEAD	TEA Domain Family Member
TET2	Ten-Eleven Translocation 2
TGF-β1	Transforming Growth Factor Beta 1
TGFβ	transforming growth factor-beta
TME	tumor microenvironment
Tregs	Regulatory T Cells
TSDR	Treg-Specific Demethylated Region
TUBA1C	Tubulin Alpha 1C
VEGF	vascular endothelial growth factor
YAP1	Yes-Associated Protein 1
15-LOX2	15-Lipoxygenase 2
ccRCC	Clear Cell Renal Cell Carcinoma
pRCC	Papillary Renal Cell Carcinoma
